# Supplementary material for: Diet and Exercise Modulate GH-IGFs Axis, Proteolytic Markers and Myogenic Regulatory Factors in Juveniles of Gilthead Sea Bream (Sparus aurata)
Source: Animals (Basel). 2021 Jul 23;11(8):2182. doi: 10.3390/ani11082182 (PMC8388392; doi:10.3390/ani11082182)

Table S1: Primers used in the Real-Time quantitative PCR analyses.

Gene	Primer sequences (5′-3′)	Ta (ºC)	Accession Number	Tissues
igf-1a	F: AGGACAGCACAGCAGCCAGACAAGAC<br>R: TTCGGACCATTGTTAGCCTCCTCTCTG	60	AY996779	L, M
igf-1ab	F: AGTCATTCATCCTTCAAGGAAGTGCATCC<br>R: TTCGGACCATTGTTAGCCTCCTCTCTG	60	EF688015	L, M
igf-1abc	F: ACAGAATGTAGGGACGGAGCGAATGGAC<br>R: TTCGGACCATTGTTAGCCTCCTCTCTG	60	EF688016	L, M
igf-2	F: TGGGATCGTAGAGGAGTGTTGT<br>R: CTGTAGAGAGGTGGCCGACA	60	AY996778	L, M
igfbp-1a	F: AGTGCGAGTCCTCTCTGGAT<br>R: TCTCTTTAAGGGCACTCGGC	60	KM522771	L, M
igf-1ra	F: AGCATCAAAGACGAACTGG<br>R: CTCCTCGCTGTAGAAGAAGC	55	KT156846	M
igf-1rb	F: GCTAATGCGAATGTGTTGG<br>R: CGTCCTTTATGCTGCTGATG	55	KT156847	L, M
ghr-1	F: ACCTGTCAGCCACCACATGA<br>R: TCGTGCAGATCTGGGTCGTA	60	AF438176	M
ghr-2	F: GAGTGAACCCGGCCTGACAG<br>R: GCGGTGGTATCTGATTCATGGT	60	AY573601	L, M
capn1	F: CCTACGAGATGAGGATGGCT<br>R: AGTTGTCAAAGTCGGCGGT	56	KF444899	M
capn2	F: ACCCACGCTCAGACGGCAAA<br>R: CGTTCCCGCTGTCATCCATCA	61	KF444900	M
capn3	F: AGAGGGTTTCAGCCTTGAGA<br>R: CGCTTTGATCTTTCTCCACA	56	ERP000874	M
capns1a	F: CGCAGATACAGCGATGAAAA<br>R: GTTTTGAAGGAACGGCACAT	56	KF444901	M
capns1b	F: ATGGACAGCGACAGCACA<br>R: AGAGGTATTTGAACTCGTGGAAG	56	ERP000874	M
ctsl	F: ACTCCTTGGGCAAACACA<br>R: CCTTGAACTTCCTCTCCGT	54	DQ875329	M
ctsda	F: CCTCCATTCACTGCTCCTTC<br>R: ACCGGATGGAAAACTCTGTG	56	AF036319	M
mafbx	F: GGTCACCTGGAGTGGAAGAA<br>R: GGTGCAACTTTCTGGGTTGT	60	ERA047531	M
murf1	F: GTGACGGCGAGGATGTGC<br>R: CTTCGGCTCCTTGGTGTCTT	60	FM145056	M

n3	F: AGACACACACTGAACCCGA<br>R: TTCCTGAAGCGAACCAGA	54	KJ524458	M
ub	F: ACTGGCAAGACCATTACCTT<br>R: TGGATGTTGTAGTCGGAAAG	54	KJ524459	M
myod1	F: TTTGAGGACCTGGACCC<br>R: CTTCTGCGTGGTGATGGA	60	AF478568.1	M
myod2	F: CACTACAGCGGGGATTCAGAC<br>R: CGTTTGCTTCTCCTGGACTC	60	AF478569	M
mrf4	F: CATCCCACAGCTTTAAAGGCA<br>R: GAGGACGCCGAAGATTCACT	60	JN034421	M
myogenin	F: CAGAGGCTGCCCAAGGTCGAG<br>R: CAGGTGCTGCCCGAACTGGGCTCG	68	EF462191	M
mstn1	F: GTACGACGTGCTGGGAGACG<br>R: CGTACGATTCGATTCGCTTG	60	AF258448.1	M
mstn2	F: ACCTGGTGAACAAAGCCAAC<br>R: TGCGGTTGAAGTAGAGCATG	60	AY046314	M
ef1a	F: CTTCAACGCTCAGGTCATCAT<br>R: GCACAGCGAAACGACCAAGGGGA	60	AF184170	L, M
rps18	F: TGACGGAAGGGCACCACCAG<br>R: AATCGCTCCACCAACTAAGAACGG	60	AY550956	L, M
tom20	F: TGTTCATCGGGTACTGCATC<br>R: TTCAGGTCTGGAAGCTTTGC	60	FM146454.1	L, M
rpl27a	F: AAGAGGAACACAACTCACTGCCCCAC<br>R: GCTTGCCTTTGCCCAGAACTTTGTAG	60	AY188520	L, M

F: forward; R: reverse; L: liver; M: muscle; Ta: annealing temperature; *igf-1a*: insulin-like growth factor 1a; *igf-1ab*: insulin-like growth factor 1ab; *igf-1abc*: insulin-like growth factor 1abc; *igf-2*: insulin-like growth factor 2; *igfbp-1a*: insulin-like growth factor binding protein 1a; *igf-1ra*: insulin-like growth factor 1 receptor a; *igf-1rb*: insulin-like growth factor 1 receptor b; *ghr-1*: growth hormone receptor 1; *ghr-2*: growth hormone receptor 2; *capn1*: calpain 1; *capn2*: calpain 2; *capn3*: calpain 3: *capns1a*: calpain subunit 1a; *capns1b*: calpain subunit 1b; *ctsl*: cathepsin l; *ctsda*: cathepsin da; *mafbx*: muscle atrophy F-box or atrogin-1; *murf1*: muscle RING finger protein 1; *n3*: proteasome type 4 subunit or PSMB4; *ub*: ubiquitin; *myod1*: myoblast determination protein 1; *myod2*: myoblast determination protein 2; *mrf4*: myogenic regulatory factor 4; *mstn1*: myostatin 1; *mstn2*: myostatin 2; *ef1a*: elongation factor 1 alpha; *rps18*: ribosomal protein s18; *tom20*: mitochondrial import receptor subunit TOM20; *rpl27a*: ribosomal protein l27a.

The supplementary figure S1 contain the original images of the Western blots and their respective total protein stains used in the present work. For each antibody, two gels were performed and the specific immunoreactive bands that were quantified are highlighted by red arrows. The distribution of the samples within each gel is the same for all of them (see the well numeration in the Capn1 images), where for each membrane, wells 1-3 correspond to VS-HE; 4-6 to SS-HP; 7-9 to VS-HP and 10-12 to SS-HE. In the case of the Capn1, the Western blots were prepared from a sections two membranes that were split to be incubated with other antibodies. For the preparation of the figures, sets of two representative bands from both the blots and the corresponding total protein stains were cropped. The cropped bands from the total protein stain used for the figures are highlighted by green arrows.

Capn1

MEMB 1 & 2

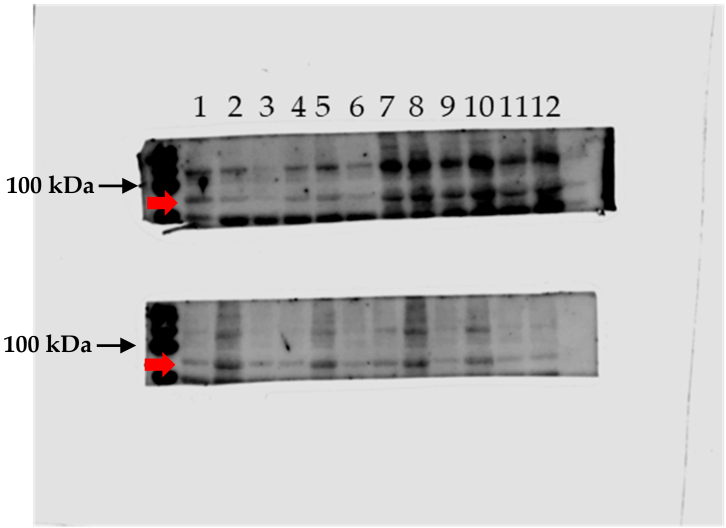

MEMB 1 TOTAL PROT

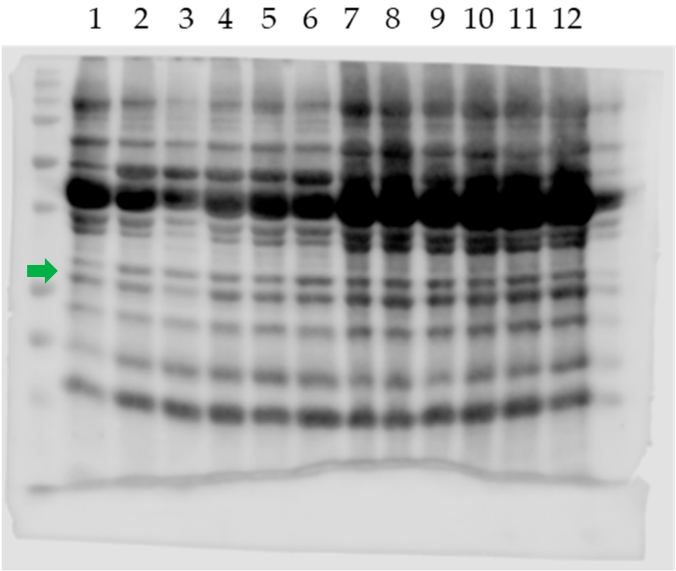

MEMB 2 TOTAL PROT

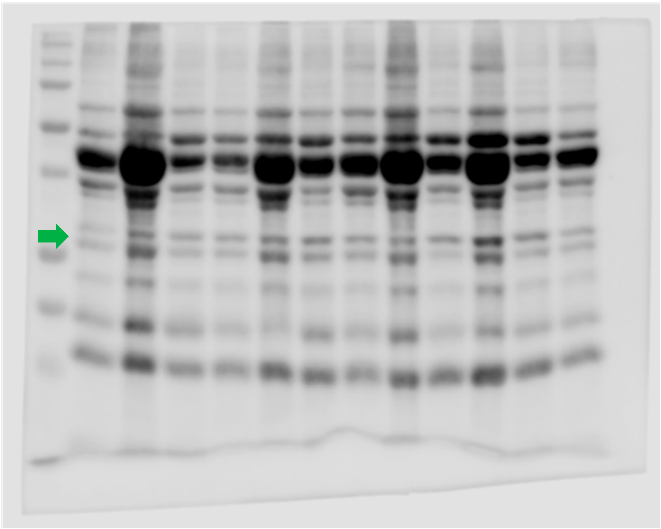

Ctsl

MEMB 1

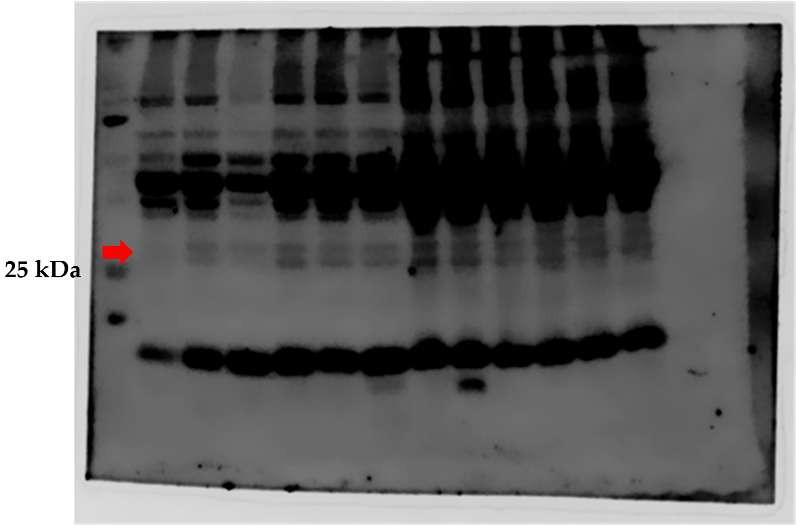

MEMB 2

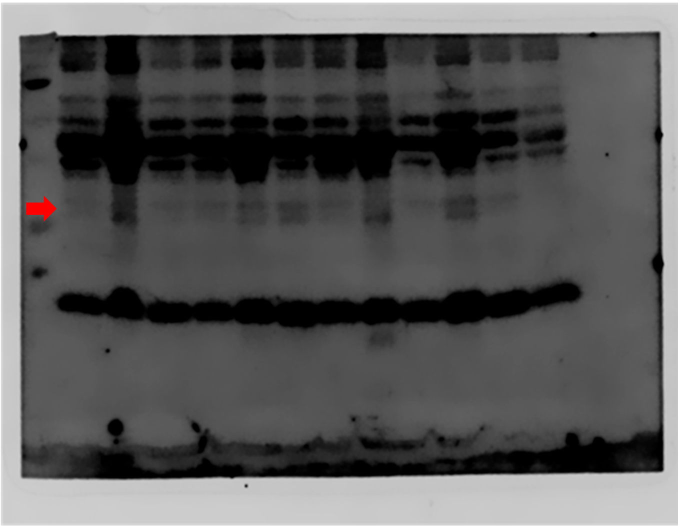

MEMB 1 TOTAL PROT

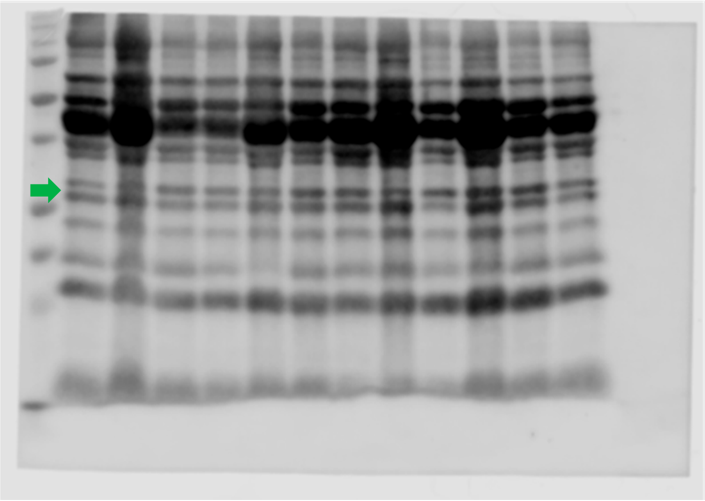

MEMB 2 TOTAL PROT

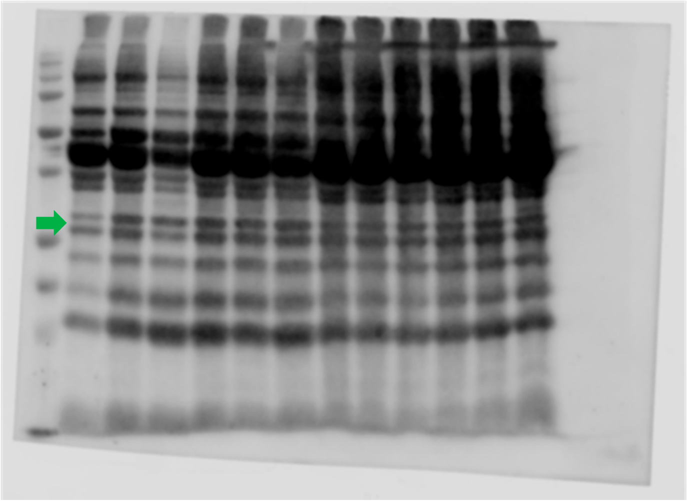

Ctsda

MEMB 1

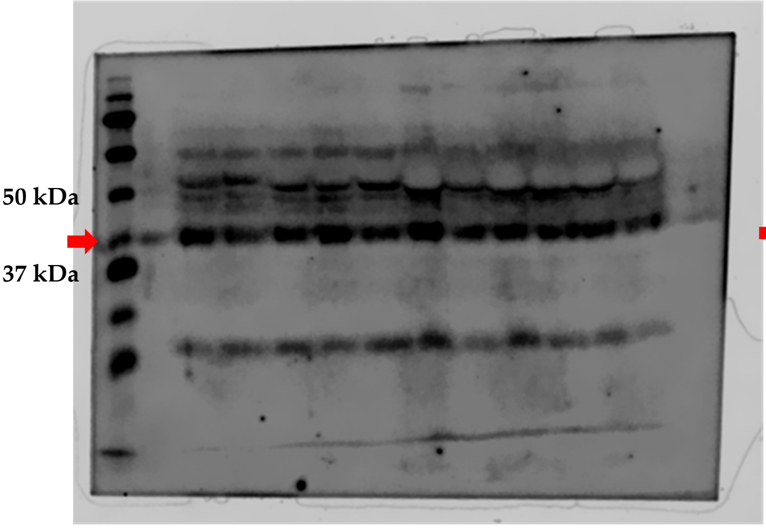

MEMB 2

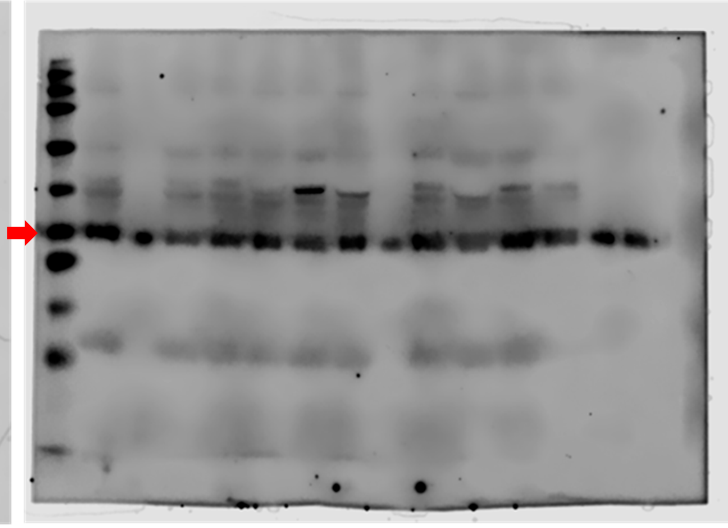

MEMB 1 TOTAL PROT

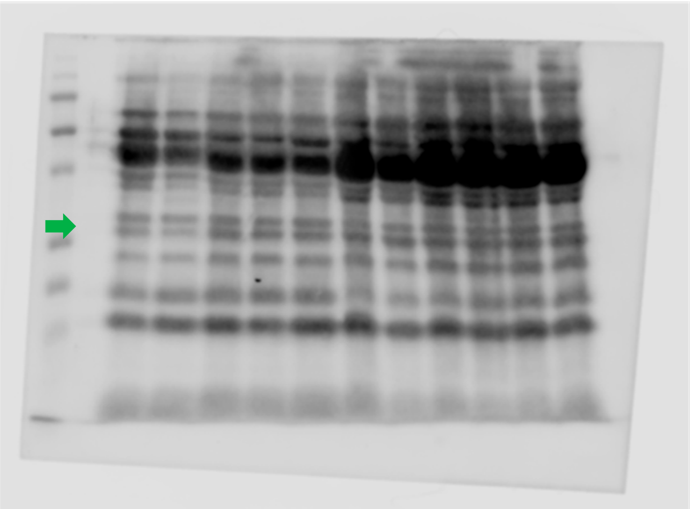

MEMB 2 TOTAL PROT

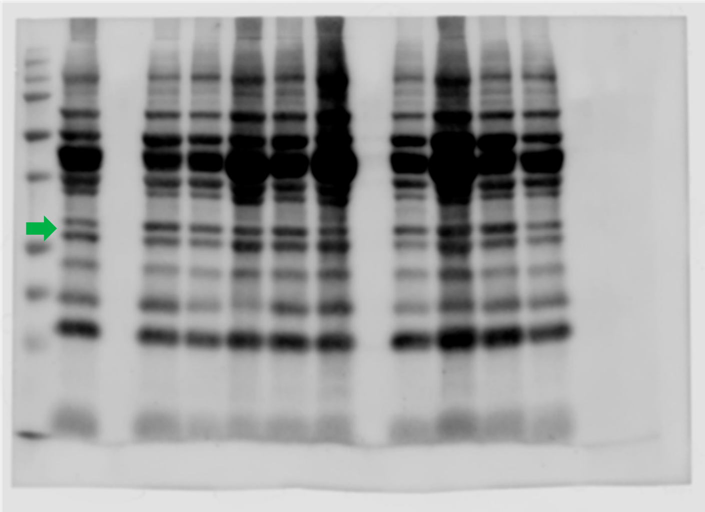

Mafbx

MEMB 1

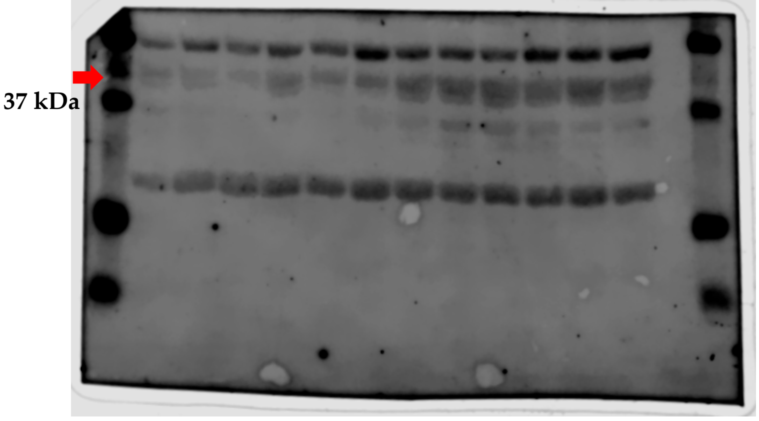

MEMB 2

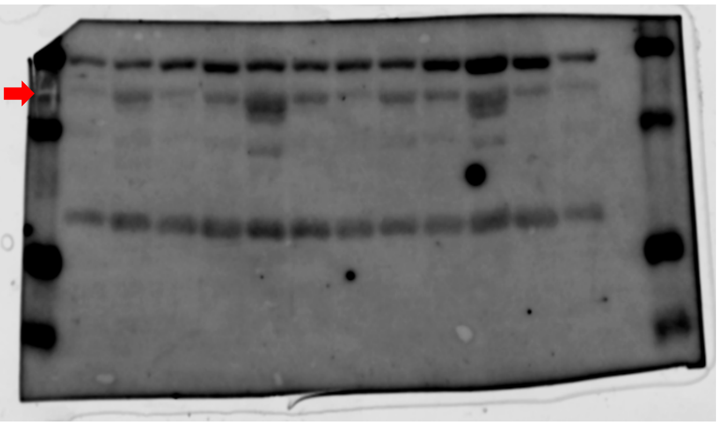

MEMB 1 TOTAL PROT

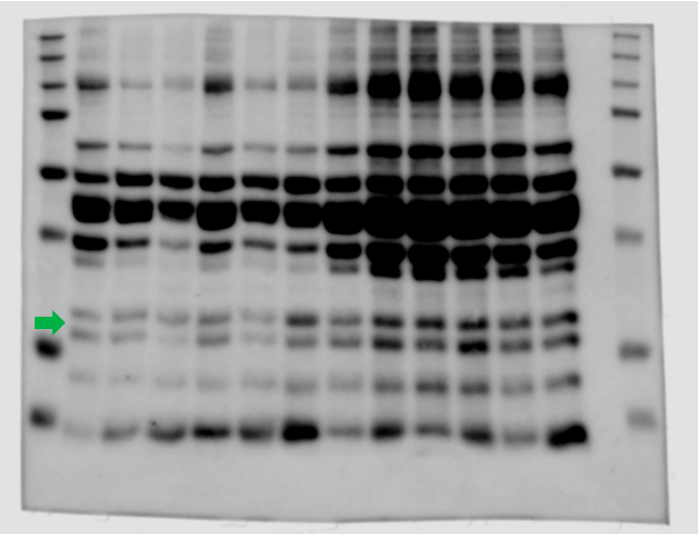

MEMB 2 TOTAL PROT

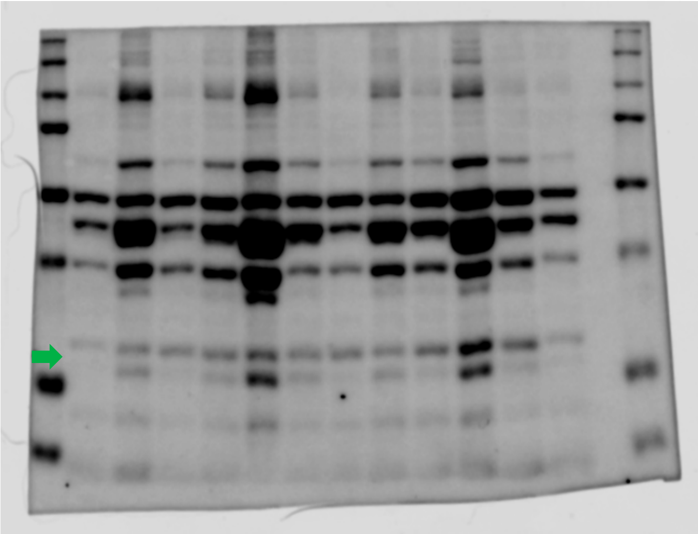

Supplement: Supplementary file 1 [file animals-11-02182-s001.zip › animals-1271150-supplementary.pdf]
